# Availability of healthier options in traditional and nontraditional rural fast-food outlets

**DOI:** 10.1186/1471-2458-8-395

**Published:** 2008-11-28

**Authors:** Jennifer S Creel, Joseph R Sharkey, Alex McIntosh, Jenna Anding, J Charles Huber

**Affiliations:** 1 Texas Healthy Aging Research Network, School of Rural Public Health, Texas A&M Health Science Center, MS 1266, College Station, TX, USA; 2 Department of Social and Behavioral Health, School of Rural Public Health, Texas A&M Health Science Center, College Station, TX, USA; 3 Department of Sociology, Texas A&M University, College Station, TX, USA; 4 Department of Nutrition and Food Science, Texas A&M University, College Station, TX, USA; 5 Department of Epidemiology and Biostatistics, School of Rural Public Health, Texas A&M Health Science Center, College Station, TX, USA

## Abstract

**Background:**

Food prepared away from home has become increasingly popular to U.S. families, and may contribute to obesity. Sales have been dominated by fast food outlets, where meals are purchased for dining away from home or in the home. Although national chain affiliated fast-food outlets are considered the main source for fast food, fast foods are increasingly available in convenience stores and supermarkets/grocery stores. In rural areas, these nontraditional fast-food outlets may provide most of the opportunities for procurement of fast foods.

**Methods:**

Using all traditional and nontraditio nal fast-food outlets identified in six counties in rural Texas, the type and number of regular and healthiermenu options were surveyed using on-site observation in all food venues that were primarily fast food, supermarket/grocery store, and convenience store and compared with 2005 Dietary Guidelines.

**Results:**

Traditional fast-food outlets represented 84 (41%) of the 205 opportunities for procurement of fast food; 109 (53.2%) were convenience stores and 12 (5.8%) supermarkets/grocery stores. Although a s imilar variety of regular breakfast and lunch/dinner entrées were available in traditional fast-food outlets and convenience stores, the variety of healthier breakfast and lunch/dinner entrées was significantly greater in fast food outlets. Compared with convenience stores, supermarkets/grocery stores provided a greater variety of regular and healthier entrées and lunch/dinner side dishes.

**Conclusion:**

Convenience stores and supermarkets/grocery stores more than double the potential access to fast foods in this rural area than traditional fast-food outlets alone; however, traditional fast food outlets offer greater opportunity for healthier fast food options than convenience stores. A complete picture of fast food environment and the availability of healthier fast food options are essential to understand environmental influences on diet and health outcomes, and identify potential targets for intervention.

## Background

Between 1977 and 1996, a dramatic shift in food sources in the U.S. was observed that reflected a significant increase in calories consumed from away-from-home versus home-prepared food.[1] Trends indicate that more Americans eat out, and today, almost 50% of the U.S. food dollar is spent at full-service and fast food restaurants[2,3]. Meals purchased away from home are playing an increasingly important role in the amount and type of foods consumed [1,4]. Household economics, opportunity costs, time constraints, and convenience appear to be major factors that influence greater reliance on food prepared outside the home [5-7].

Fast-food places have surpassed full-service restaurants as the largest source of away-from-home foods[1], which may explain why many researchers believe that consumption of fast-food items is a contributor to obesity[8,9]. Current investigations point to the increased availability of energy-dense foods as a major contributor to energy imbalance and obesity [10-17]. Fast food is considered to be low-cost (and almost resistant to inflation)[18], energy-dense, low in several important nutrients, and high in calories, fat, and cholesterol[1,17]. Increased consumption of fast food is associated with lower intake of fruits and vegetables, increased body weight, increased intake of carbonated beverages, and lower consumption of milk and grains[8,17,19,20].

Some full-service and fast-food restaurants have recently added healthier options to menus as well as menu identification of healthier options; however, there have been few studies to examine such offeringsin fast food restaurants [21-25]. Although identifying healthier options and providing nutrition information on the menu appears to be an emerging trend within the food industry, a great deal of variability exists between food outlets and available nutrition information[4,22,23,26].

While these studies provide nascent insight into the availability of healthier fast-food options in traditional, "big chain" fast-food restaurants in urban areas, little is known about availability in rural areas in the United States. Rural areas in the United States are increasing in population, especially in minority population [27,28]. At the same time, there is a greater prevalence of obesity among adults and children [29-31] and limited access to supermarkets, which provide larger selections of healthy foods [32-34]. Further, there is little understanding of the extent to which convenience stores, supermarkets, and mass merchandisers have added fast foods to their primary business as they seek new sales opportunities and increase the access of fast foods to consumers. This "channel blurring" has created nontraditional fast-food outlets, where fast food items are sold by retail stores in which the primary business is not fast food [6].

This study expands our understanding of the fast-food environment by: 1) identifying all opportunities for the procurement of fast-food entrées and side dishes in a six-county rural region of Texas using ground-truth methods; and 2) determining the extent to which a variety of regular and healthier fast-food options was associated with the type of primary business – traditional fast food outlet, convenience store, and supermarket/grocery.

## Methods

### Rural setting and sample

Data were obtained from the 2006 Brazos Valley Food Environment Project (BVFEP) for six rural counties in the central Brazos Valley region of Texas (land area of 11,567 km^2^). The six rural counties comprise, along with one urban county, a planning region created by the legislature. The region's boundaries were based upon such characteristics as geographic features, economic market areas, labor markets, and commuting patterns[35]. The BVFEP, which was approved by the Institutional Review Board at Texas A&M University, is an ongoing examination of the food environment using ground-truth methods. Details on the counties and ground-truth methods for identifying all food stores and food service places have been reported elsewhere [33]. Briefly, all highways (Interstate, U.S., and State), farm-to-market roads, and city or town streets and roads within the six-county area were systematically driven; a "windshield survey" completed; and on-site geographic coordinates determined to identify, classify, and locate the position of all food stores and food service places [33]. All counties were revisited to ensure the completeness of the data. The study sample included all retail locations that provided an opportunity for the procurement of fast food.

### Data collection procedure

A two-part observational survey instrument was developed, based on prior restaurant audits, recommendations from the 2005 Dietary Guidelines for Americans, and input from local Registered Dietitians [36-38]. The first part recorded site information, such as store type, store hours, store exterior (parking lot and building), condition of the parking lot, ads or promotions identifying fast food, ads or promotions for healthy foods, store interior, store size (e.g., number of booths and tables), and number of registers. The second part of the survey instrument included an assessment of menu items (e.g., entrées, side dishes, beverages, and desserts) including availability of healthier options, identification of nutritional information, and preparation methods [39,40].

### Measures

#### Outlet Type

Using data from the BVFEP, all traditional fast food outlets, supermarkets/grocery stores, and convenience stores were assessed for availability of fast-food items. In addition to traditional fast food outlets, only those supermarkets/grocery stores (*n *= 12) and convenience stores (*n *= 109) that offered fast food items were included in the sample. Using the North American Industry Classification System as a guide, we defined the outlets based on their primary business[41]. Traditional fast food outlets are limited-service restaurants that provide the sale of quick service foods that are ready for immediate consumption on premises, taken out or delivered to the customer's location, and where customers pay before eating. These do not include snack and nonalcoholic beverage bars[41,42]. Convenience stores or food marts (with or without fuel pumps) are primarily engaged in retailing a limited line of good that generally includes milk, bread, soda, and snacks. This can either be a convenience store or a gasoline station setting. Supermarkets or grocery stores are primarily engaged in retailing a general line of food; fresh fruits and vegetables; and fresh and prepared meats, fish, and poultry[41]. After the pre-test assessment and modification, data were collected on-site over a four-week period (August to September 2006), and entered from hard copy into a relational database.

#### Breakfast Entrée

Four breakfast entrées were identified: 1) breakfast sandwich, 2) breakfast taco, 3) breakfast meals, and 4) breakfast pastry. An entrée was considered to be healthier when at least one of the following options were available: lean meat, 100% whole wheat/whole grain bread, low-fat cheese, 100% whole wheat/whole grain tortilla, eggs without cheese, or pastry made of 100% whole wheat/whole grain or low-fat. A regular breakfast entrée variety score was calculated by summing the number of breakfast entrées (range 0–4); a healthier breakfast entrée variety score was calculated by summing the number of healthier breakfast entrées (range 0–1).

#### Lunch/Dinner Entrée

Eleven lunch/dinner entrées were identified with healthier options including: grilled meat, chicken, fish, or other cooked meats; 100% whole wheat/whole grain bun, pizza crust, tortilla, or wrap; no breading on chicken, fish, ormeats; lean cuts of other cooked meats, cold cuts, or meat salads; low-fat cheese; low-fat or fat-free dressing; baked chips; brown rice; low-fat sauce or no sauce option; or no added fat, such as cheese or bacon. A healthier lunch/dinner entrée would be classified as having a healthier option when at least one healthier option was identified. A regular lunch/dinner entrée variety score was calculated by summing the number of lunch/dinner entrées (range 0–10); a healthier lunch/dinner entrée variety score was calculated by summing the number of healthier lunch/dinner entrées (range 0–5).

#### Lunch/Dinner Side Dishes

The availability of side dishes with healthier options included: fruit (either without added fat or sugar, or 100% fruit juice); vegetables that were either steamed/roasted or not fried; potatoes with at least one of the following options – baked, no fat added, or low-fat options; soup identified as either low fat or reduced sodium; baked chips; potato salad with low-fat dressing; chili with either lean meat or turkey; corn either without fat or without sauce; or coleslaw with low-fat dressing. A side dish was classified as having a healthier option when at least one healthier option was identified. A regular lunch/dinner side dish variety score was calculated by summing the number of lunch/dinner side dishes (range 0–5); a healthier lunch/dinner side dish variety score was calculated by summing the number of healthier lunch/dinner side dishes (range 0–2).

#### National Chain Affiliation

A binary variable, national chain affiliation, identified fast food outlets as being affiliated with a national chain (n = 56).

### Statistical analysis

All statistical analyses were performed using Stata Statistical Software Release 9 (Stata Corporation, College Station, TX, 2005). The availability of a regular and healthier breakfast entrée, lunch/dinner entrée, and lunch/dinner side dish were calculated by type of primary business for the entire sample. Summary scores were calculated for regular breakfast entrée variety, healthier breakfast entrée, regular lunch/dinner entrée, healthier lunch/dinner entrée, regular lunch/dinner side dish, healthier lunch/dinner side dish. The difference in variety by type of primary business was assessed by using one-way analysis of variance, with Bonferroni correction for multiple comparisons.

Multivariable regressions were used to examine the correlation of type of primary business and national chain affiliation with each of the six variety scores. Since each variable consisted of "count" data, standard procedures were used to determine if each of the outcome variables of interest may be reasonably described by a Poisson distribution. The results of these procedures indicate that none of the six outcome variables can be reasonablydescribed by a Poisson distribution. Therefore the data were analyzed using ordinal logistic regression with robust (White-Huber-corrected) Standard Errors. Six multivariable ordinal regression models were individually fitted to determine the relationship of type of primary business (i.e., traditional fast food outlet, convenience store, supermarket/grocery store) and national chain affiliation with: 1) regular breakfast entrée variety, 2) healthier breakfast entrée variety, 3) regular lunch/dinner entrée variety, 4) healthier lunch/dinner entrée variety, 5) regular lunch/dinner side dish variety, and 6) healthier lunch/dinner side dish variety. Statistical significance was set at *p *< 0.05.

## Results

BVFEP data included 261 fast food outlets, convenience stores, and grocery stores/supermarkets. At the time of the fast food survey, 11 stores were no longer in business, and 45 stores did not sell fast food items (34 convenience stores and 11 supermarket/grocery stores). This provided a final sample of 205 opportunities for the procurement of fast food: 84 (41%) fast food outlets, 109 (53.2%) convenience stores, and 12 (5.8%) supermarkets/grocery stores. More than 65 percent of the 84 fast food outlets were national chain brand (n = 56). Table 1 shows the availability of regular and healthier breakfast entrées, overall and by type of primary business. Among the business that offered fast foods, 46.8% (*n *= 96) did not market a breakfast entrée (data not shown). Breakfast sandwiches were most frequently available, followed by breakfast tacos. Very few healthy options were available for breakfast sandwiches, tacos, or pastry.

**Table 1:** Menu identification of regular and healthier breakfast entrées, overall and by type of primary business

	Overall (n = 205) % (n)	Traditional Fast Food (n = 84) % (n)	Convenience (n = 109) % (n)	Supermarket/Grocery (n = 12) % (n)
Individual breakfast entrée				
Breakfast sandwich*	47.8 (98)	33.3 (28)	56.0 (61)	75.0 (9)
Healthier option^†^	5.1 (5)	12.8 (5)	0	0
Breakfast taco*	33.2 (68)	34.5 (30)	28.4 (31)	58.3 (7)
Healthier option^†^	5.9 (4)	6.7 (2)	1.8 (2)	0
Breakfast meal*	14.6 (30)	17.9 (15)	8.3 (9)	50.0 (6)
Healthier option^†^	96.7 (29)	100.0 (15)	88.9 (8)	100.0 (6)
Breakfast pastry*	15.1 (31)	19.0 (16)	11.9 (13)	16.7 (2)
Healthier option^†^	0	0	0	0

*Percent of overall (combined), traditional fast food outlets, convenience stores, or supermarket/grocery stores

^†^Percent of overall, traditional fast food outlets, convenience stores, or supermarkets/grocery stores that offered a specific breakfast entrée

The availability of regular and healthier lunch/dinner entrées are shown in Table 2. More than 78% of locations offering chicken served it as deep fried (91.2% of fast food outlets, 65.7% of convenience stores, and 90.9% of supermarkets/grocery stores); a similar percentage also offered chicken that was not fried or breaded. Deep frying was customary method of preparation for fish (78%). Entrée types providing the greatest amounts of healthier options were chicken and entrée salads.

**Table 2:** Menu identification of regular and healthier lunch/dinner entrées, overall and by type of primary business

	Overall (n = 205) % (n)	Traditional Fast Food (n = 84) % (n)	Convenience (n = 109) % (n)	Supermarket/Grocery (n = 12) % (n)
Individual lunch/dinner entrée				
Hamburger*	53.2 (109)	50.0 (42)	56.0 (61)	50.0 (6)
Healthier option^†^	0	0	0	0
Chicken*	67.3 (138)	67.9 (57)	64.2 (70)	91.7 (11)
Healthier option^†^	65.2 (90)	80.7 (46)	30.3 (33)	81.8 (11)
Fish*	24.4 (50)	27.4 (23)	16.5 (18)	75.00 (9)
Healthier option^†^	0	0	0	0
Other cooked meats*	48.3 (99)	41.7 (35)	51.4 (56)	66.7 (8)
Healthier option^†^	4.0 (4)	0	5.4 (3)	12.5 (1)
Cold cuts/meat salads*	45.4 (93)	21.4 (18)	60.5 (66)	75.0 (9)
Healthier option^†^	14.0 (13)	61.1 (11)	3.0 (2)	0
Pizza*	19.0 (39)	19.0 (16)	20.2 (22)	8.3 (1)
Healthier option^†^	35.9 (14)	62.5 (10)	18.2 (4)	0
Mexican food*	46.8 (96)	33.3 (28)	58.7 (64)	33.3 (4)
Healthier option^†^	14.6 (14)	32.1 (9)	7.8 (5)	0
Asian food*	18.5 (38)	3.6 (3)	24.8 (27)	66.7 (8)
Healthier option^†^	0	0	0	0
Salad as entrée*	34.6 (71)	54.8 (46)	15.6 (17)	66.7 (8)
Healthier option^†^	77.5 (55)	91.3 (42)	47.1 (8)	62.5 (5)
Hot dogs*	41.5 (85)	26.2 (22)	52.3 (57)	50.0 (6)
Healthier option^†^	0	0	0	0
Wrap sandwich*	16.6 (34)	32.1 (27)	4.6 (5)	16.7 (2)
Healthier option^†^	67.6 (23)	74.1 (20)	20.0 (1)	100.0 (2)

*Percent of overall (combined), traditional fast food outlets, convenience stores, or supermarket/grocery stores

^†^Percent of overall, traditional fast food outlets, convenience stores, or supermarkets/grocery stores that offered a specific lunch/dinner entrée

Side dishes were available in 125 (61%) of all locations. Table 3 shows the availability of regular and healthier side dishes. Overall, potatoes were the most frequently side dish, followed vegetables. The availability of healthier options for beverages and desserts was also surveyed (results not shown). More than 94% of all locations offered sugar-free soft drinks, while reduced or non-fat milk was available at 10 fast food outlets (11.9%), and low-fat or reduced sugar ice cream was available at 14.3% (*n *= 12) fast food outlets and 1.8% (*n *= 2) convenience stores.

**Table 3:** Menu identification of regular and healthier lunch/dinner side dishes, overall and by type of primary business

	Overall (n = 205) % (n)	Traditional Fast Food (n = 84) % (n)	Convenience (n = 109) % (n)	Supermarket/Grocery (n = 12) % (n)
Individual side dish				
Fruit*	2.4 (6)	5.9 (5)	0.9 (1)	0
Healthier option^†^	83.3 (5)	100.0 (5)	0	0
Vegetables*	21.0 (43)	20.2 (17)	15.6 (17)	75.0 (9)
Healthier option^†^	53.5 (23)	47.1 (8)	35.3 (6)	100.0 (9)
Potato*	50.7 (104)	65.5 (55)	35.8 (39)	83.3 (10)
Healthier option^†^	11.5 (12)	9.1 (5)	12.8 (5)	20.0 (2)
Soup*	2.9 (6)	5.9 (5)	0	8.3 (1)
Healthier option^†^	0	0	0	0
Chips*	14.6 (30)	25.0 (21)	8.3 (9)	0
Healthier option^†^	36.7 (11)	52.4 (11)	0	0
Potato salad*	12.2 (25)	10.7 (9)	6.4 (7)	75.0 (9)
Healthier option^†^	4.0 (1)	11.1 (1)	0	0
Chili*	1.5 (3)	2.4 (2)	0	8.3 (1)
Healthier option^†^	0	0	0	0
Corn*	10.2 (21)	11.9 (10)	6.4 (7)	33.3 (4)
Healthier option^†^	80.9 (17)	70.0 (7)	85.7 (6)	100.0 (4)
Cole slaw*	15.1 (31)	17.9 (15)	7.3 (8)	66.7 (8)
Healthier option^†^	0	0	0	0

*Percent of overall (combined), traditional fast food outlets, convenience stores, or supermarket/grocery stores

^†^Percent of overall, traditional fast food outlets, convenience stores, or supermarkets/grocery stores that offered a specific lunch/dinner side dish

Difference in variety scores among the three types of primary businesses are shown in Table 4. Supermarket/grocery stores provided a greater variety of regular entrées (breakfast and lunch/dinner) and side dishes than traditional fast food outlets or convenience stores. Convenience stores offered less variety in healthier breakfast and lunch/dinner entrées than either traditional fast food outlets or supermarket/grocery stores.

**Table 4:** Comparison of regular and healthier entrée and side dish variety scores by type of primary business*

	Traditional Fast Food (n = 84)	Convenience (n = 109)	Supermarket/Grocery (n = 12)
Breakfast entrée variety scores			
Regular breakfast entrée variety	1.06 ± 1.41	1.05 ± 1.06	2.00 ± 1.54^§‡^
Healthier breakfast entrée variety^†^	0.63 ± 0.55	0.15 ± 0.36^¶^	0.67 ± 0.50^§^
			
Lunch/dinner entrée variety scores			
Regular lunch/dinner entrée variety	3.77 ± 2.01	4.25 ± 2.33	6.00 ± 2.09^§‡^
Healthier lunch/dinner entrée variety^†^	2.0 ± 1.33	0.53 ± 0.79^¶^	1.42 ± 1.08^§^
			
Lunch/dinner side dish variety scores			
Regular lunch/dinner side dishes variety	1.65 ± 1.33	0.81 ± 1.17^¶^	3.50 ± 1.78^§‡^
Healthier lunch/dinner side dish variety^†^	0.52 ± 0.71	0.39 ± 0.69	1.50 ± 0.85^§‡^

Values are mean ± standard deviation

*One-way analysis of variance, with Bonferroni adjustment for multiple comparisons

^†^Healthier variety score values for businesses that market regular entrée or side dish

^‡ ^Difference of means between supermarket/grocery stores and traditional fast food outlets (*p *< 0.05)

^§^Difference of means between supermarket/grocery stores and convenience stores (*p *< 0.05)

^¶ ^Difference of means between convenience stores and traditional fast food outlets (*p *< 0.05)

The results from multivariable ordinal logistic analyses for regular and healthier variety of breakfast entrées, lunch/dinner entrées, and lunch/dinner side dishes are shown in Table 5. Data are presented as Odds Ratios (OR) and 95% Confidence Intervals (CI) using White-Huber-corrected SE. Compared with fast food outlets, convenience stores and supermarket/grocery stores were more likely to have a greater variety of regular entrées and side dishes. When it came to healthier entrées or side dishes, traditional fast food outlets offered a greater variety of healthier breakfast entrées, healthier lunch/dinner entrées, and healthier lunch/dinner side dishes.

**Table 5:** Odds ratios and 95% CI from ordinal logistic regression models correlating type of food outlet with increased variety of regular and healthier breakfast entrées, lunch/dinner entrées, and lunch/dinner side dishes

	Breakfast Entrée Variety		Lunch/dinner entrée variety		Lunch/dinner side dish variety	
	Regular breakfast entrée variety	Healthier breakfast entrée variety	Regular lunch/dinner entrée variety	Healthier lunch/dinner entrée variety	Regular lunch/dinner side dish variety	Healthier lunch/dinner side dish variety
	OR (95% CI)	OR (95% CI)	OR (95% CI)	OR (95% CI)	OR (95% CI)	OR (95% CI)

Convenience store*	1.5 (0.64, 3.3)	0.30^‡ ^(0.10,0.85)	2.3^‡ ^(1.03, 5.3)	0.24^¶ ^(0.11, 0.48)	0.17^¶ ^(0.07, 0.40)	0.23^¶ ^(0.10, 0.55)
Supermarket/grocery store*	5.2^‡ ^(1.1, 24.2)	2.9 (0.72, 12.1)	10.6^¶ ^(2.8, 39.7)	1.02 (0.28, 3.7)	8.4^§ ^(1.7, 42.1)	2.3 (0.53, 9.8)
National chain affiliation^†^	1.2 (0.44, 3.1)	0.98 (0.35, 2.7)	2.1 (0.95, 4.9)	2.3 (0.99, 5.5)	0.59 (0.27, 1.3)	0.63 (0.27, 1.5)

Pseudo *R*^2^of model	0.013	0.081	0.017	0.086	0.087	0.060
Significance of *χ*^2 ^in model	0.191	0.002	0.006	<0.0001	<0.0001	<0.001

* Referent is traditional fast food outlet

^†^Referent is no national chain affiliation

^‡^*P *< 0.05

^§^*P *< 0.010

^¶^*P *< 0.001

Interestingly, having an affiliation with a national chain was not associated with variety in any of the regular or healthier entrées or side dishes.

## Discussion

Although the foodservices market recognizes several trends in foodservice, such as healthier menu options and blurring the channels by marketing products outside a company's primary product area[6,43], little is known about the availability of healthier fast-food options beyond a few traditional national chain brandsin urban areas [4,21-24]. This is the first study, to our knowledge, that describes the availability of healthier options for breakfast and lunch/dinner entrées and lunch/dinner side dishes in traditional and nontraditional fast-food opportunitiesin a large rural area. Three key findings warrant further examination: 1) in this study sample, more opportunities exist for the procurement of fast-food entrées and side dishes from convenience stores and supermarket/groceries than from the traditional fast-food outlet where the primary business is fast food; 2)supermarket/grocery stores had a greater variety of entrées and side dishes than traditional fast-food outlets or convenience stores; and 3) convenience stores offered significantly less variety of healthier breakfast and lunch/dinner entrées and lunch/dinner side dishes than did traditional fast food outlets.

Based on utilization of ground-truth methods (e.g., driving all major roads and conducting on-site surveys) to identify opportunities for procuring fast food [33], almost 60% of the fast food opportunities in the six rural counties were provided by nontraditional fast food outlets where the primary business was as convenience stores or supermarkets/grocery stores. This is important, given that the preponderance of research on access to fast food focused on traditional fast food outlets, especially the national chains [44-48]. As important as away-from-home foods are to dietary intake, restricting the measurement of fast foods to traditional fast food locations may overlook a substantial portion of the fast food available within a given food environment. The decision to purchase food away from home or prepare food at home is weighed by the consumer based on cost in time and money, which commodity is of greater value, and which method of food acquisition allows retention of the commodities[6,49,50]. For the individual who makes food choices based on travel time and money, fast food typically offers a quick meal at an inexpensive price. Travel time as a commodity may be of even greater value in rural environments where the travel distance between all destinations (including grocery stores for the purchase of raw goods) may be great.

A number of factors might explain the larger number of nontraditional fast food outlets in the survey area, compared with traditional fast-food outlets. Convenience stores, which have built their business on fast service and longer hours of operation, understand the consumer's need for convenient shopping, especially one-stop shopping[51]. With increased costs and competition from other retail channels, the addition of fast food helps convenience store operators attract and hold customers on a daily basis, thereby increasing revenues[43]. Concomitantly, rural residents face added transportation costs; have better spatial access to convenience stores [33]; and thus may demand more services in one location. Additionally, supermarkets and grocery stores face the encroachment of other retail channels, such as dollar stores, mass merchandisers, and drugstores[52]. As a result, supermarkets and groceries seek to expand their offerings and provide the consumer with convenient, appealing foods and a reason to shop more frequently.

Interestingly, supermarket/grocery stores were consistently offered a greater variety of regular fast-food breakfast entrées, lunch/dinner entrées, or lunch/dinner side dishes than either fast food outlets or convenience stores. However, when controlling for other primary businesses and national chain affiliation, convenience stores more likely to offer a greater variety of regular lunch/dinner entrées and lower variety of healthier breakfast entrées, lunch/dinner entrées or side dishes than traditional fast-food outlets. To residents in these six rural counties, convenience stores provide best access (nearest location) to food items[33]. The results of this study extend those findings to suggest best access to less variety of healthier fast-food entrées. Furthermore, in addition to traditional fast-food outlets, convenience stores should be targeted for expansion of healthier food offerings. While the findings of this study offer insight into the availability of healthier food options at all stores selling fast food within rural areas, further investigation would likely identify potential strategies for increasing healthier options within these stores.

There are several limitations that require mention. First, we were unable to assess exact nutritional information. Due to a lack of nutritional information on menus, it is difficult to assess whether a menu item identified as "low fat" or "light" would actually be considered a healthier option according to recommendations of the Dietary Guidelines[37]. Second, the availability of healthier food items may have been underestimated where a menu did not identify a healthier option as being healthier (e.g., turkey breast or deli chicken breast). Third, we are unable to report test-retest or inter-rater reliability results. Fourth, data did not capture time of day for the assessment and the potentialunderestimation of breakfast offerings when data were collected later in the day and breakfast menus, signs, or food items were not visible. We expect this to be a factor more in a convenience store or supermarket/grocery store than in a fast food outlet. Future qualitative work will include interviews with owners/managers and observations of the stores during business hours to identify barriers and facilitators for making additional healthier options available within all types of fast-food opportunities and communicating this to the public. Finally, full-service restaurants are now increasing their marketing of take-out foods[53]. Future work will include an assessment of healthier options in take-out foods from full-service restaurants.

## Conclusion

Despite these limitations, this study furthers our knowledge about availability of opportunities for the procurement of fast food in a rural environment. By using ground-truthed methods, this study reduced the potential for misrepresentation based on data from publicly or commercially available list of food outlets[33]. Food options and information made available to the consumer play a large role in the selection of food within in a food store location. This study highlights the variability in the variety of healthier fast-food options among traditional fast-food outlets, convenience stores, and supermarkets/grocery stores, all selling fast food in a rural environment. While the influence of consumer demand for various food options cannot be ignored, the lack of available healthier options should be considered as an intervention point for improving the health status of rural populations. Food intake is directly related to weight status, which can be associated with negative health outcomes. The environment plays a pivotal role in an individual's food acquisition (and thus intake), as a consumer can only purchase and consume those foods that are available [54-56].

## Competing interests

The authors declare that they have no competing interests.

## Authors' contributions

JC developed the original idea for assessing the availability healthy options. JS worked with JC on the development of the observational instrument and the protocol for collection of data. JC wrote the first draft of the paper. JH assisted with multivariate analyses. JS, AM, JA, and JH provided comments and proposed revisions. All authors have read and approved the final manuscript.

## Pre-publication history

The pre-publication history for this paper can be accessed here:


